# Neutrophil-to-lymphocyte and neutrophil-to-platelet ratios for predicting 28-day mortality in sepsis: A retrospective cohort study

**DOI:** 10.1371/journal.pone.0348268

**Published:** 2026-05-13

**Authors:** Jianchun Wei, Xinjie Huang, Zhilian Wang, Hailing Zhang

**Affiliations:** 1 Department of Emergency Medicine, Aviation General Hospital, Beijing, China; 2 Department of General Surgery, Beijing Children’s Hospital, Capital Medical University, Beijing, China; Children's National Hospital, George Washington University, UNITED STATES OF AMERICA

## Abstract

**Objective:**

This study aimed to evaluate the prognostic value of the neutrophil-to-lymphocyte ratio (NLR) and neutrophil-to-platelet ratio (NPR), alone and in combination with procalcitonin (PCT), for predicting 28‑day mortality in patients with sepsis.

**Methods:**

In this single‑center retrospective cohort study, 395 adult sepsis patients admitted to the emergency department between 2020 and 2025 were included. NLR and NPR were calculated from the first complete blood count obtained within 24 hours of initial emergency department presentation. Univariable Cox regression was used for preliminary screening. The C-index was used to compare the discriminative ability of traditional inflammatory markers (PCT, CRP, and WBC) to select a reference biomarker. Multivariable Cox regression and restricted cubic spline (RCS) analysis were performed to evaluate the linear and non-linear associations of NLR and NPR with mortality. To assess whether adding NLR or NPR provided incremental prognostic value to PCT, multivariable Cox models with interaction terms (PCT × NLR and PCT × NPR) were constructed. LASSO regression was applied for variable selection, and VIF was used to assess multicollinearity.

**Results:**

The overall 28‑day mortality was 38.2%. Neither NLR nor NPR outperformed PCT in predicting 28-day mortality, and none of the three biomarkers showed significant independent associations with mortality after adjusting for confounders (PCT: adjusted HR per IQR = 1.02, 95% CI: 0.94–1.11, *p* = 0.605; NLR: HR = 1.01, 95% CI: 1.00–1.02, *p* = 0.235; NPR: HR = 1.00, 95% CI: 1.00–1.01, *p* = 0.378). Restricted cubic spline (RCS) curves confirmed no non-linear associations or threshold effects for NLR and NPR. Multivariable Cox regression with interaction terms showed that adding NLR or NPR to PCT did not provide additional prognostic value beyond PCT alone (PCT × NLR: HR = 1.00, 95% CI: 0.97–1.03, *p* = 0.854; PCT × NPR: HR = 0.97, 95% CI: 0.92–1.01, *p* = 0.121).

**Conclusion:**

Neither NLR nor NPR alone, nor their combination with PCT, improved 28-day mortality prediction in this elderly sepsis cohort, suggesting that a single early measurement may be insufficient for sepsis prognostication. Future studies should explore serial measurements and integrate multiple pathophysiological dimensions. The low cost and wide availability of NLR and NPR still support their potential role within a multi-parameter panel, particularly in resource-limited settings.

## Introduction

Sepsis is a life-threatening clinical syndrome characterized by organ dysfunction resulting from a dysregulated host response to infection. As a major global health challenge, it affects millions annually and imposes a substantial disease and economic burden [1,2]. Epidemiological data reveal considerable geographic and systemic variation in its incidence. In high-income countries with robust healthcare systems, the estimated incidence of hospital-treated adult sepsis is 189 per 100,000 person-years (95% confidence interval, CI: 133–267), a figure that may have risen in the past decade to 276 per 100,000 person-years (95% CI: 189–403), with some regional reports indicating even higher rates. Among patients severe enough to require intensive care unit (ICU) admission, the incidence is approximately 58 per 100,000 person-years (95% CI: 42–81), accompanied by an in-hospital mortality rate as high as 41.9% (95% CI: 36.2–47.7) [3]. However, these statistics are predominantly derived from high-income nations. In contrast, low- and middle-income countries, which bear the majority of the global population and a high infection burden, critically lack population-based epidemiological studies. Consequently, the true global burden of sepsis is likely substantially underestimated, particularly in resource-limited settings where the need for effective, accessible prognostic tools is most acute [4].

Early diagnosis of sepsis and the initiation of standardized treatment before patients require ICU admission have been proven to significantly reduce mortality [5]. However, achieving early diagnosis still faces persistent challenges. On the one hand, the typical clinical manifestations of sepsis—such as fever, tachycardia, and tachypnea—lack specificity and are easily confused with common infections or inflammatory conditions. On the other hand, there is generally insufficient awareness among both healthcare professionals and the public, leading to low vigilance and often resulting in delayed presentation and recognition.

Blood cultures play a central diagnostic role in detecting sepsis, particularly when bloodstream infection (bacteremia or fungemia) is suspected. However, blood cultures suffer from long turnaround time, low sensitivity, requirement of specific culture conditions, and difficulties in growing fastidious organisms [6–8]. Currently, there is still a lack of an ideal biomarker or rapid bedside detection tool that can accurately identify sepsis in a timely manner. This issue is particularly prominent in populations such as infants, young children, and the elderly, who may exhibit subtle symptoms yet experience rapid disease progression [9]. Therefore, exploring and validating potential sepsis recognition tools or biomarkers that may enable early detection, are low-cost, and can be easily obtained from routine laboratory tests has become a worthwhile pursuit for improving the prognosis of sepsis patients.

The neutrophil-to-lymphocyte ratio (NLR), a biomarker derived from routine complete blood counts, offers the advantages of easy accessibility, low cost, and high reproducibility. NLR has been extensively studied for prognostic assessment and risk stratification in various diseases, including cardiovascular conditions [10], sepsis [11,12], and malignancies [13]. In sepsis, immune dysregulation is characterized by neutrophilia and lymphopenia. As a composite index, an elevated NLR reflects both excessive inflammation and immune suppression, offering prognostic value in clinical practice.

The neutrophil-to-platelet ratio (NPR) is an inflammatory-coagulation composite biomarker derived from routine complete blood counts. It integrates information from neutrophil-driven acute inflammatory responses and platelet-mediated immune-coagulation interactions. NPR has demonstrated significant prognostic value across various inflammation-associated conditions, including ST‑segment elevation myocardial infarction (STEMI) [14], infective endocarditis [15], and sepsis [16]. An increased NPR reflects both acute inflammation and immunothrombosis, providing integrated inflammatory and coagulation information for early risk stratification and therapeutic decision‑making.

In summary, NLR and NPR, as readily accessible markers of systemic inflammation and immune status, have attracted increasing attention for their prognostic value. However, a systematic evaluation of these markers for 28‑day mortality in sepsis patients is still lacking. Therefore, through a retrospective cohort analysis, this study aims to: (1) compare the predictive value of NLR or NPR alone versus traditional inflammatory markers for 28‑day mortality in sepsis patients; and (2) determine whether adding NLR or NPR to traditional inflammatory markers provides additional prognostic value beyond these markers alone, with the goal of providing more efficient and clinically accessible composite indicators for early risk stratification.

## Materials and methods

### Study design and participants

This retrospective cohort study consecutively enrolled 395 sepsis patients admitted to the Emergency Department of Aviation General Hospital between 03/04/2020 and 27/07/2025. The study protocol was reviewed and approved by the Ethics Committee of Aviation General Hospital on 19/01/2026. Subsequently, the data for research purposes were accessed and collected between 20/01/2026 and 26/01/2026.

Inclusion criteria:

1. Aged 18 years or older;
2. Meeting the Sepsis-3.0 diagnostic criteria, specifically the presence of a documented or suspected infection with an associated increase in the Sequential Organ Failure Assessment (SOFA) score of 2 points or greater [1,2];
3. Availability of at least one complete blood count (CBC) record within 24 hours after admission to the emergency department.

Exclusion criteria:

1. Hospital stay < 24 hours (due to self-discharge or very early death resulting in insufficient data);
2. Known hematologic malignancies (e.g., leukemia, lymphoma) or myelodysplastic syndromes, as these conditions may significantly and unpredictably affect peripheral blood cell counts.
3. History of chemotherapy or radiotherapy within the past 3 months；
4. Pregnancy or lactation;
5. Clinically significant missing data that precludes calculation or analysis.

### Ethical approval and consent to participate

This retrospective medical record study involving human participants was reviewed and approved by the Ethics Committee of Aviation General Hospital. The approval number is HK LL-2026-001-01. The requirement for informed consent was waived by the ethics committee because the data were analyzed anonymously and retrospectively.

### Data collection and parameter calculation

A comprehensive set of baseline variables was collected for each patient, comprising the following categories:

1. Demographics and Clinical Status: Age, sex, and primary clinical manifestations.
2. Comorbidities: A history of hypertension, coronary artery disease, diabetes mellitus, and cerebrovascular disease (including ischemic stroke or intracerebral hemorrhage).
3. Infection Characteristics: primary infection site (categorized as respiratory tract, urinary tract, abdominal, or other, e.g., skin and soft tissue, central nervous system, bloodstream) and pathogen identification results (Gram‑positive, Gram‑negative, or other pathogens including fungi, viruses and atypical pathogens).
4. Laboratory Parameters: White blood cell count (WBC), absolute neutrophil count (NEUT), absolute lymphocyte count (LYM), platelet count (PLT), C-reactive protein (CRP), procalcitonin (PCT), lactate (Lac), prothrombin time (PT), blood glucose (Glu), serum creatinine (Cr), alanine aminotransferase (ALT), aspartate aminotransferase (AST), and total bilirubin (TBil).
5. Derived Parameters and Scores: NLR and NPR were calculated from the differential complete blood count. Disease severity was assessed using the Sequential Organ Failure Assessment (SOFA) score.

### Sampling time windows

All baseline parameters were obtained from the first available clinical and laboratory records within 24 hours after admission to the emergency department.

### Definitions of Key Variables

All ratios and scores were derived from the first complete blood count obtained at emergency department presentation.

- NLR: Calculated as the absolute neutrophil count divided by the absolute lymphocyte count.

$$\begin{array}{c} Formula:\;NLR=Neutrophil\;count\;\left(\times {\text{1}\text{0}}^{\text{9}}/L\right)/Lymphocyte\;count\;\left(\times {\text{1}\text{0}}^{\text{9}}/L\right) \end{array}$$

- NPR: Calculated as the absolute neutrophil count divided by the platelet count.

$$\begin{array}{c} Formula:\;NPR=Neutrophil\;count\;\left(\times {\text{1}\text{0}}^{\text{9}}/L\right)/Platelet\;count\;\left(\times {\text{1}\text{0}}^{\text{9}}/L\right) \end{array}$$

### Statistical analysis

Statistical analyses were performed using SPSS version 26.0 (IBM Corp., Armonk, NY, USA) and R software version 4.5.1 (R Foundation for Statistical Computing, Vienna, Austria). Continuous variables, presented as median (interquartile range, IQR), were compared using the Mann–Whitney U test. Categorical variables, expressed as n (%), were compared using the χ² test or Fisher’s exact test, as appropriate (Table 1).

**Table 1:** Baseline characteristics and clinical outcomes of septic patients stratified by 28-day survival status.

Characteristic	Total (n = 395)	Survivors (n = 244)	Non-survivors (n = 151)	Statistical Value	*p*-value
**Demographics**					
Age, years	81 (70,87)	79 (69, 86)	82 (72, 88)	−2.244	0.025
Male sex, n (%)	220 (55.7)	139 (57.0)	81(53.6)	0.418	0.518
**Comorbidities, n (%)**					
Hypertension	198 (50.1)	133 (54.5)	65 (43.0)	4.902	0.027
Coronary artery disease	124 (31.4)	77 (31.6)	47 (31.1)	0.008	0.928
Diabetes mellitus	126 (31.9)	77 (31.6)	49 (32.5)	0.034	0.853
Cerebrovascular disease ^a^	134 (33.9)	90 (36.9)	44 (29.1)	2.497	0.114
**Site of Infection, n (%)**				6.950	0.074
Respiratory tract	235 (59.5)	137 (56.1)	98 (64.9)		
Urinary tract	57 (14.4)	41 (16.8)	16 (10.6)		
Abdominal	82 (20.8)	56 (23.0)	26 (17.2)		
Others sites ^b^	21 (5.3)	10 (4.1)	11 (7.3)		
**Pathogens, n (%)**					
Any pathogen detected	298 (75.4)	183 (75.0)	115 (76.2)	0.068	0.795
Gram-positive	95 (24.1)	66 (27.0)	29 (19.2)	3.142	0.076
Gram-negative	234 (59.2)	154 (63.1)	80 (53.0)	3.968	0.046
Other pathogens ^c^	75 (19.0)	39 (16.0)	36 (23.8)	3.744	0.053
**Disease Severity Scores**					
SOFA score, points	7 (4, 9)	6 (4,8)	8 (6,10)	−6.028	<0.001
**Infection-Related Parameters**					
WBC	11.4 (8.0, 16.6)	10.9 (7.8, 16.4)	11.6 (8.6, 17.4)	−1.111	0.267
CRP	55.8 (15.0, 127.8)	48.0 (10.0, 115.0)	73.0 (23.5, 148.5)	−2.942	0.003
PCT	0.98 (0.21, 7.84)	0.6 (0.1, 6.1)	1.9 (0.4, 11.9)	−3.075	0.002
**Clinical and Laboratory Parameters**					
Lac	2.2 (1.2, 4.0)	1.8 (1.0, 3.0)	2.9 (1.5, 5.6)	−4.802	<0.001
PT	13.4 (12.3, 14.9)	13.0 (12.1, 14.1)	14.4 (12.9, 15.9)	−6.123	<0.001
Cr	105.0 (66.2, 179.9)	95.9 (63.2, 146.3)	130.1 (74.3, 258.7)	−3.405	<0.001
ALT	21.5 (14.0, 40.0)	20.0 (14.5, 36.8)	23.0 (13.6, 43.4)	−0.417	0.677
AST	31.7 (22.5, 51.8)	28.6 (21.4, 42.7)	37.2 (25.1, 59.9)	−3.648	<0.001
TBil	17.3 (12.7, 24.5)	17.3 (12.5, 23.8)	17.3 (13.0, 26.1)	−1.003	0.316
Glu	9.0 (6.8, 12.1)	9.0 (6.8, 11.9)	9.3 (6.7, 12.5)	−0.219	0.826
**Hematological Parameters and Derived Indices**					
NEUT	9.4 (6.4, 13.8)	9.2 (6.2, 13.8)	10.2 (6.9, 14.4)	−1.289	0.197
LYM	0.93 (0.55, 1.41)	0.95 (0.54, 1.50)	0.90 (0.59, 1.33)	−0.800	0.424
PLT	210 (148, 288)	216 (147, 291)	205 (148, 274)	−0.989	0.323
NLR	10.14 (5.56, 18.55)	9.76 (5.15, 18.36)	12.06 (6.36, 19.14)	−1.335	0.182
NPR	0.046 (0.031, 0.070)	0.044 (0.028, 0.069)	0.051 (0.034, 0.073)	−1.755	0.079

**Note:** Data are presented as median (interquartile range) for continuous variables and number (%) for categorical variables. Group comparisons were performed using the Mann‑Whitney U test for continuous variables and the χ² test for categorical variables, as appropriate.

**Abbreviations:** ALT, alanine aminotransferase; AST, aspartate aminotransferase; Cr, creatinine; CRP, C-reactive protein; Glu, glucose; Lac, lactate; LYM, lymphocyte count; NEUT, neutrophil count; NLR, neutrophil-to-lymphocyte ratio; NPR, neutrophil-to-platelet ratio; PCT, procalcitonin; PLT, platelet count; PT, prothrombin time; SOFA, Sequential Organ Failure Assessment; TBil, total bilirubin; WBC, white blood cell.

**Units:** WBC, NEUT, LYM, PLT: × 10⁹/L; CRP: mg/L; PCT: ng/mL; Lac: mmol/L; PT: s; Cr: μmol/L; ALT, AST: U/L; TBil: μmol/L; Glu: mmol/L.

^a^ Cerebrovascular disease included a history of ischemic stroke or intracerebral hemorrhage.

^b^ Other infection sites included skin and soft tissue, central nervous system, and bloodstream infections.

^c^ Other pathogens included fungi, viruses, and atypical pathogens.

Univariable Cox proportional hazards regression was performed using SPSS to screen for factors associated with 28-day mortality (Tables 2 and 3).

**Table 2:** Univariate Cox regression analysis of continuous variables associated with 28-day mortality in sepsis patients.

Characteristic	Median (Q1–Q3)	IQR range	Crude HR (per IQR)	95% CI	*p*-value
**Demographics**					
Age	81 (70- 87)	17	1.13	0.91–1.41	0.268
**Disease Severity Score**					
SOFA score	7 (4, 9)	5	1.94	1.53–2.47	< 0.001
**Infection-Related Parameters**					
WBC	11.4 (8.0,16.6)	8.6	1.06	0.90–1.26	0.477
CRP	55.8 (15.0, 127.8)	112.8	1.39	1.14–1.70	0.001
PCT	0.98 (0.21, 7.84)	7.63	1.07	1.00–1.14	0.066
**Clinical and Laboratory Parameters**					
Lac	2.2 (1.2, 4.0)	2.8	1.35	1.22–1.50	< 0.001
PT	13.4 (12.3, 14.9)	2.6	1.03	1.00–1.07	0.083
Cr	105.0 (66.2, 179.9)	113.7	1.18	1.09–1.27	< 0.001
ALT	21.5 (14.0, 40.0)	26.0	1.00	0.96–1.04	0.808
AST	31.7 (22.5, 51.8)	29.3	1.01	0.99–1.03	0.239
TBil	17.3 (12.7, 24.5)	11.8	1.06	1.02–1.10	0.006
Glu	9.0 (6.8, 12.1)	5.3	1.00	0.90–1.10	0.979
**Hematological Parameters and Derived Indices**					
NEUT	9.4 (6.4, 13.8)	7.4	1.00	1.00–1.01	0.450
LYM	0.93 (0.55, 1.41)	0.86	1.02	0.93–1.12	0.672
PLT	210 (148, 288)	140	0.96	0.79–1.17	0.692
NLR	10.14 (5.56, 18.55)	12.99	1.00	1.00–1.01	0.368
NPR	0.046 (0.031, 0.070)	0.039	1.00	1.00–1.01	0.414

**Note:** Data are presented as crude HR and 95% CI from univariable Cox proportional hazards regression.

**Abbreviations:** ALT, alanine aminotransferase; AST, aspartate aminotransferase; CI, confidence interval; Cr, creatinine; CRP, C-reactive protein; Glu, glucose; HR, hazard ratio; IQR, interquartile range; Lac, lactate; LYM, lymphocyte count; NEUT, neutrophil count; NLR, neutrophil-to-lymphocyte ratio; NPR, neutrophil-to-platelet ratio; PCT, procalcitonin; PLT, platelet count; PT, prothrombin time; SOFA, Sequential Organ Failure Assessment; TBil, total bilirubin; WBC, white blood cell.

**Units:** WBC, NEUT, LYM, PLT: × 10⁹/L; CRP: mg/L; PCT: ng/mL; Lac: mmol/L; PT: s; Cr: μmol/L; ALT, AST: U/L; TBil: μmol/L; Glu: mmol/L.

**Table 3:** Univariate Cox regression analysis of categorical variables associated with 28-day mortality in sepsis patients.

Characteristic	Category	Crude HR	95% CI	*p*-value
**Demographics**				
Sex	Male vs. Female	0.90	0.65–1.24	0.514
**Comorbidities**				
Hypertension	Yes vs. No	0.65	0.47-0.89	0.008
Coronary artery disease	Yes vs. No	0.98	0.69-1.38	0.894
Diabetes mellitus	Yes vs. No	0.98	0.70-1.38	0.922
Cerebrovascular disease^a^	Yes vs. No	0.67	0.47-0.95	0.024
**Site of Infection**				
Respiratory tract	Yes vs. others	1.17	0.83-1.63	0.370
Urinary tract	Yes vs. others	0.74	0.44-1.24	0.245
Abdominal	Yes vs. others	0.90	0.59-1.38	0.638
Others sites^b^	Yes vs. others	1.22	0.66-2.25	0.529
**Pathogens**				
Gram-positive	vs. no Gram-positive infection	0.61	0.41-0.92	0.018
Gram-negative	vs. no Gram-negative infection	0.55	0.40-0.76	<0.001
Other pathogens^c^	vs. no other pathogen infection	1.36	0.93-1.97	0.109

**Note:** Data are presented as crude HR and 95% CI from univariable Cox proportional hazards regression.

**Abbreviations:** CI, confidence interval; HR, hazard ratio.

^a^ Cerebrovascular disease included a history of ischemic stroke or intracerebral hemorrhage.

^b^ Other infection sites included skin and soft tissue, central nervous system, and bloodstream infections.

^c^ Other pathogens included fungi, viruses, and atypical pathogens.

**Fig 1:**
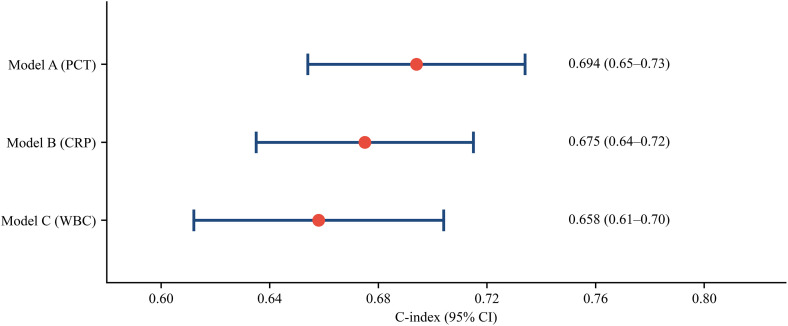
C-Index comparison of traditional inflammatory markers for predicting 28-day mortality.

To select a representative traditional inflammatory marker for subsequent comparative analyses, we evaluated the discriminative ability of three candidate markers—PCT, CRP, and WBC—using C-index, adjusted for core clinical baseline variables (age, SOFA score, and Lac). C-index calculations and forest plots were generated using R software (Table 4, Fig 1).

**Table 4:** Discriminative ability of traditional inflammatory markers for 28-day mortality.

Model	Variables Included	C-index	95% CI
Model A	Baseline* + PCT	0.694	0.65–0.73
Model B	Baseline* + CRP	0.675	0.64–0.72
Model C	Baseline* + WBC	0.658	0.61–0.70

**Note:** Data are presented as C-index with 95% CI. Higher C-index indicates better discriminative ability.

**Abbreviations:** CI, confidence interval; CRP, C-reactive protein; PCT, procalcitonin; WBC, white blood cell.

* Baseline variables included age, SOFA score, and Lac.

Multivariable Cox regression was then performed using SPSS to evaluate the prognostic value of individual inflammatory markers (PCT, NLR, and NPR) for 28-day mortality, adjusted for age, SOFA score, and Lac (Table 5).

**Table 5:** Multivariable Cox regression analysis of PCT, NLR, and NPR for 28-day mortality.

Biomarker	Adjusted HR (per IQR)	95% CI	p-value
PCT	1.02	0.94–1.11	0.605
NLR	1.01	1.00–1.02	0.235
NPR	1.00	1.00–1.01	0.378

**Note:** Adjusted HRs were derived from multivariable Cox proportional hazards regression models. All models were adjusted for age, SOFA score, and Lac. HRs are expressed per IQR increase for clinical interpretability.

**Abbreviations:** CI, confidence interval; HR, hazard ratio; IQR, interquartile range; NLR, neutrophil-to-lymphocyte ratio; NPR, neutrophil-to-platelet ratio; PCT, procalcitonin.

**Fig 2:**
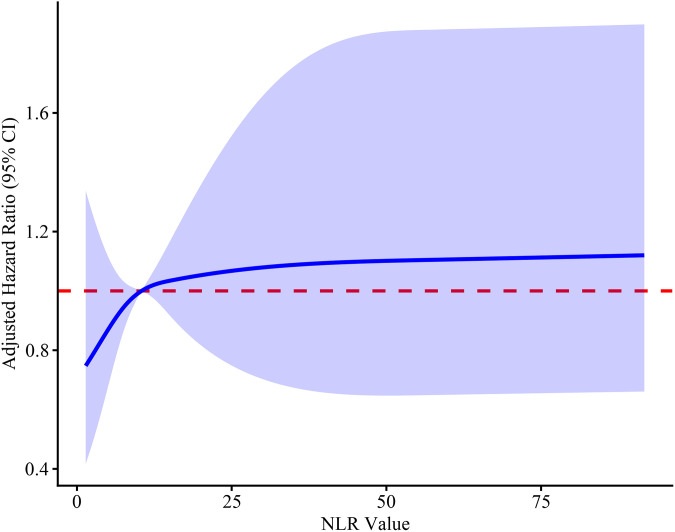
Restricted cubic spline curve for NLR. The solid line represents the adjusted hazard ratio, and the shaded area represents the 95% confidence interval. Models were adjusted for age, SOFA score, and Lac (set at their medians: age = 81 years, SOFA = 7, Lac = 2.2 mmol/L).

**Fig 3:**
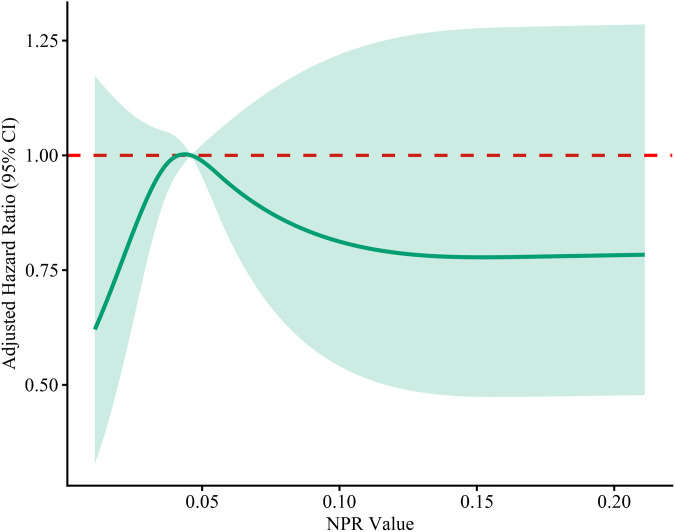
Restricted cubic spline curve for NPR. The solid line represents the adjusted hazard ratio, and the shaded area represents the 95% confidence interval. Models were adjusted for age, SOFA score, and Lac (set at their medians: age = 81 years, SOFA = 7, Lac = 2.2 mmol/L).

Restricted cubic spline (RCS) analysis was performed using R software to explore non-linear associations of NLR and NPR with 28-day mortality, adjusted for the same covariates (age, SOFA score, and Lac) (Figs 2 and 3).

**Fig 4:**
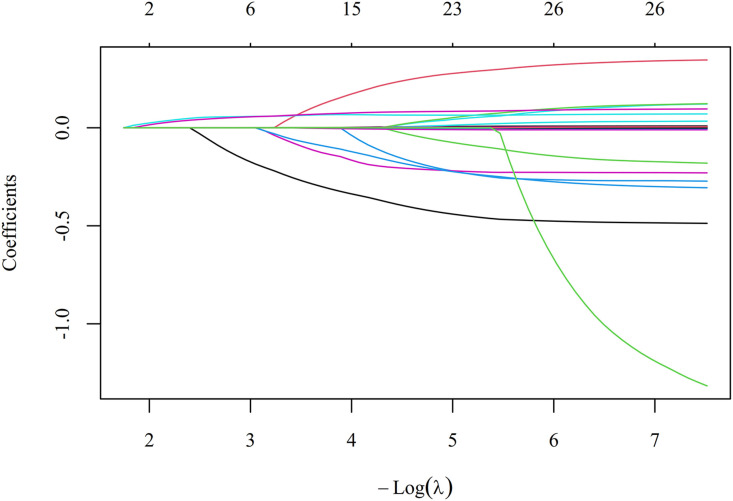
LASSO coefficient paths. Each colored line represents the coefficient trajectory of a candidate predictor as the penalty parameter λ increases. As λ increases, coefficients shrink toward zero. The vertical dashed line indicates the optimal λ selected by 10-fold cross-validation.

**Fig 5:**
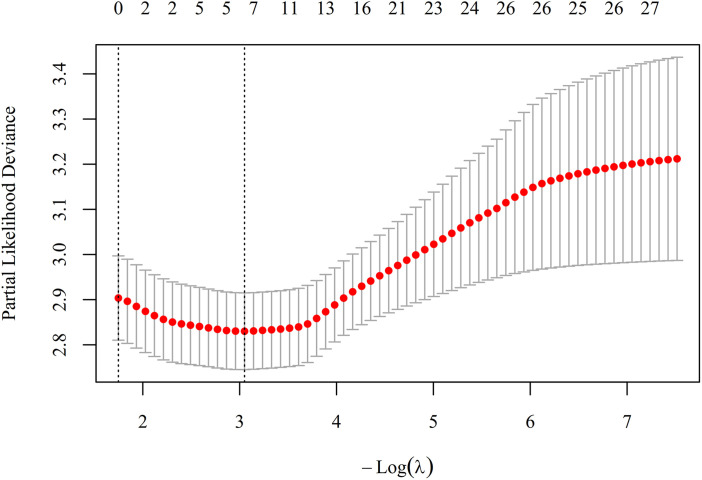
LASSO cross-validation curve. The x-axis shows log(λ), and the y-axis shows the partial likelihood deviance. The left dashed line (λ. min) identifies the λ with minimum deviance; the right dashed line (λ.1se) identifies a simpler model within one standard error of the minimum.

To avoid overfitting given the modest number of events (151 deaths) and multiple candidate predictors, least absolute shrinkage and selection operator (LASSO) Cox regression with 10-fold cross-validation was conducted using R software to identify the most robust set of core covariates (Figs 4 and 5, Table 6). Multicollinearity was assessed using variance inflation factor (VIF) in SPSS, with VIF < 5 indicating no severe multicollinearity (Table 7).

**Table 6:** LASSO-derived coefficients for selected covariates.

Variable	Coefficient
Hypertension	–0.185
SOFA score	0.058
Lac	0.057
CRP	0.001
Cerebrovascular disease	−3.5 × 10 ⁻ ⁴
Cr	3.1 × 10 ⁻ ⁴

**Note:** Coefficients were derived from LASSO Cox regression with 10-fold cross-validation using the λ. min criterion.

**Abbreviations:** CRP, C-reactive protein; Cr, creatinine; Lac, lactate; LASSO, least absolute shrinkage and selection operator; SOFA, Sequential Organ Failure Assessment.

**Table 7:** VIF for variables in the final multivariable models.

Variable	Model 1 (PCT + NLR)	Model 2 (PCT + NPR)
PCT	1.217	1.218
NLR	1.008	—
NPR	—	1.007
Hypertension	1.041	1.042
SOFA score	1.248	1.249
Lac	1.213	1.211
CRP	1.179	1.177

**Note:** VIF values below 5 indicate no severe multicollinearity.

**Abbreviations:** CRP, C-reactive protein; NLR, neutrophil-to-lymphocyte ratio; NPR, neutrophil-to-platelet ratio; PCT, procalcitonin; SOFA, Sequential Organ Failure Assessment; VIF, variance inflation factor.

Finally, multivariable Cox proportional hazards models were constructed using SPSS to evaluate the independent prognostic value of PCT combined with NLR or NPR. PCT and NLR (or NPR) were entered as continuous variables with interaction terms, adjusted for LASSO-selected covariates (hypertension, SOFA score, Lac, and CRP). All continuous variables were scaled per IQR increase for clinical interpretability (Table 8). The events per variable (EPV) was calculated to assess model stability.

**Table 8:** Multivariable Cox regression analysis of PCT combined with NLR or NPR for 28-day mortality.

Model	Variable	Adjusted HR	95% CI	p-value
Model 1 (PCT + NLR)	PCT	0.99	0.88–1.11	0.812
	NLR	1.01	1.00–1.02	0.223
	PCT × NLR	1.00	0.97–1.03	0.854
Model 2 (PCT + NPR)	PCT	1.06	0.93–1.20	0.382
	NPR	1.01	1.00–1.01	0.269
	PCT × NPR	0.97	0.92–1.01	0.121

**Note:** Both models were adjusted for hypertension, SOFA score, CRP, and lactate. HRs are expressed per IQR increase for clinical interpretability. PCT × NLR and PCT × NPR represent the interaction terms between PCT and NLR or NPR, respectively.

**Abbreviations:** CI, confidence interval; CRP, C-reactive protein; HR, hazard ratio; IQR, interquartile range; NLR, neutrophil-to-lymphocyte ratio; NPR, neutrophil-to-platelet ratio; PCT, procalcitonin.

A two‑tailed *p* < 0.05 was considered statistically significant.

## Results

This retrospective study comprised 395 adult patients with sepsis. The overall cohort had a median age of 81 (70–87) years and was predominantly male (55.7%). According to their 28-day survival status, patients were stratified into two groups: survivors (n = 244, 61.8%) and non-survivors (n = 151, 38.2%).

The baseline comparison revealed that non-survivors presented with significantly greater organ dysfunction, as quantified by SOFA score 8 (6–10) vs. 6 (4–8), *p* < 0.001. Age was significantly higher in non‑survivors (median 82 years) compared to survivors (median 79 years, *p* = 0.025). No statistically significant differences were found between the groups regarding sex distribution or the prevalence of other major comorbidities such as coronary artery disease, diabetes, or cerebrovascular disease.

Notably, the microbiological profile differed between the groups. While the overall pathogen detection rate was comparable, the proportion of infections attributed to Gram-negative bacteria was significantly lower in non-survivors than in survivors (53.0% vs. 63.1%; *p* = 0.046). Conversely, non-survivors showed a trend toward a higher prevalence of infections caused by fungi, viruses, or atypical pathogens (23.8% vs. 16.0%; *p* = 0.053). The respiratory tract was the most common infection site across the entire cohort (59.5%), with a numerically higher prevalence in non-survivors (64.9% vs. 56.1%), though this difference did not reach statistical significance for the overall distribution of infection sites (*p* = 0.074).

Critically, the hematological indices of primary interest—NLR and NPR—were numerically higher in non-survivors than in survivors, demonstrating a consistent upward trend (NLR: 12.06 vs. 9.76, *p* = 0.182; NPR: 0.051 vs. 0.044, *p* = 0.079). These preliminary findings highlight the potential prognostic value of these routinely available composite markers and justify the subsequent in-depth correlation and predictive analyses conducted in this study (Table 1).

Univariable Cox regression identified several factors associated with 28-day mortality (Tables 2 and 3). The analysis yielded hazard ratios (HRs), which quantify the association between each factor and mortality risk.

Among continuous variables (Table 2), the primary biomarkers of interest—NLR and NPR—showed no significant linear association with mortality (NLR per IQR: crude HR = 1.00, 95% CI: 1.00–1.01, *p* = 0.368; NPR per IQR: crude HR = 1.00, 95% CI: 1.00–1.01, *p* = 0.414), suggesting the absence of a simple dose-response relationship. In contrast, higher SOFA score (per IQR: crude HR = 1.94, 95% CI: 1.53–2.47, *p* < 0.001), CRP (per IQR: crude HR = 1.39, 95% CI: 1.14–1.70, *p* = 0.001), Lac (per IQR: crude HR = 1.35, 95% CI: 1.22–1.50, *p* < 0.001), Cr (per IQR: crude HR = 1.18, 95% CI: 1.09–1.27, *p* < 0.001), and Tbil (per IQR: crude HR = 1.06, 95% CI: 1.02–1.10, *p* = 0.006) were significantly associated with increased mortality. No statistically significant associations were observed for age, WBC, PCT, PT, ALT, AST, glucose, neutrophil count, lymphocyte count, or platelet count.

Among categorical variables (Table 3), Gram-positive infection (crude HR = 0.61, 95% CI: 0.41–0.92, *p* = 0.018) and Gram-negative infection (crude HR = 0.55, 95% CI: 0.40–0.76, *p* < 0.001) were associated with lower mortality, possibly reflecting early empirical broad-spectrum antibiotic use. No statistically significant associations were observed for sex, other infection sites, or other pathogens.

To select a traditional inflammatory indicator as a benchmark for subsequent comparisons with the novel indices (NLR and NPR), we evaluated the clinical utility of traditional inflammatory indicators and compared the discriminative ability (C-index) of models incorporating PCT, CRP, and WBC alongside core clinical baseline variables. As shown in Table 4, Model A (baseline+ PCT) demonstrated the highest discriminative performance with a C-index of 0.694 (95% CI: 0.65–0.73), followed by Model B (baseline + CRP) with a C-index of 0.675 (95% CI: 0.64–0.72), and Model C (baseline + WBC) with a C-index of 0.658 (95% CI: 0.61–0.70). In terms of calibration, the Hosmer-Lemeshow test yielded p-values of 0.063, 0.209, and 0.069 for Models A, B, and C, respectively, all exceeding 0.05, indicating good calibration. Therefore, based on its superior clinical discrimination, PCT was selected as the optimal reference biomarker for further comparative analyses of the composite indices (NLR and NPR).

To evaluate the prognostic value of individual inflammatory markers for 28-day mortality, we performed multivariable Cox regression assuming linear effects (Table 5). As shown in Table 5, after adjusting for age, SOFA score, and Lac, none of the biomarkers showed a significant linear association with 28-day mortality (PCT: adjusted HR per IQR = 1.02, 95% CI: 0.94–1.11, *p* = 0.605; NLR: adjusted HR per IQR = 1.01, 95% CI: 1.00–1.02, *p* = 0.235; NPR: adjusted HR per IQR = 1.00, 95% CI: 1.00–1.01, *p* = 0.378), indicating no significant linear association.

To further explore whether non-linear relationships or threshold effects existed, we performed RCS analysis for each biomarker separately (Figs 2 and 3). The RCS curves demonstrated that the 95% confidence intervals crossed the reference line (HR = 1) across the entire range of values, confirming the absence of non-linear associations or threshold effects.

Given that neither NLR nor NPR exhibited independent linear or non-linear associations with 28-day mortality when evaluated alone (Table 5, Figs 2 and 3), we next investigated whether adding these composite indices to PCT could improve its predictive performance. However, given the modest number of events (151 deaths) and multiple candidate predictors, we first performed LASSO Cox regression with 10-fold cross-validation to avoid overfitting and to identify the most robust set of core covariates (Figs 4 and 5). Based on the LASSO selection (λ. min criterion), six covariates with non-zero coefficients were retained: hypertension, cerebrovascular disease, SOFA score, Lac, CRP and Cr (Table 6). After comprehensive evaluation considering clinical relevance and effect magnitude, we retained four covariates with meaningful effect sizes: hypertension, SOFA score, Lac and CRP. Cerebrovascular disease and Cr were excluded due to their negligible coefficients (close to zero), and Cr was additionally excluded because SOFA score already incorporates renal function assessment. These four covariates, together with PCT as the reference inflammatory marker (based on Table 4), were used as the adjustment set in subsequent multivariable Cox analyses.

Before constructing the final multivariable Cox models, we assessed multicollinearity among the selected covariates using the VIF, with values below 5 indicating no severe multicollinearity (Table 7).

After confirming no severe multicollinearity, we constructed the final multivariable Cox models to evaluate the independent prognostic value of PCT combined with NLR or NPR. The EPV was 21.6 (151/7) for each model, exceeding the recommended threshold of 10, indicating adequate model stability and no overfitting (Table 8). As shown in Table 8, when evaluated as continuous variables scaled by IQR, adding NLR to PCT did not provide additional prognostic value beyond PCT alone (PCT × NLR: HR = 1.00, 95% CI: 0.97–1.03, *p* = 0.854). Similarly, adding NPR to PCT also showed no significant interaction (PCT × NPR: HR = 0.97, 95% CI: 0.92–1.01, *p* = 0.121). These findings demonstrate that neither NLR nor NPR improved mortality prediction when combined with PCT.

## Discussion

Sepsis is a critical medical emergency characterized by a life-threatening organ dysfunction due to a dysregulated host response to infection. Its high mortality and substantial global burden necessitate urgent improvements in early management. Each hour of delay in initiating appropriate antimicrobial therapy is associated with a significant increase in mortality risk, underscoring that early recognition and accurate risk stratification are paramount to improving patient outcomes. This fundamental clinical challenge highlights the imperative to develop and validate accessible prognostic tools that can be deployed at the initial point of care to guide timely and targeted interventions [1,3,17,18].

The pathophysiological foundation of sepsis lies in a complex and often dysregulated immune response. Neutrophils, as frontline defenders, release neutrophil extracellular traps (NETs) to ensnare pathogens; however, this mechanism can paradoxically exacerbate tissue injury and microvascular damage through protease release, illustrating their dual role in both defense and immunopathology [19]. Meanwhile, lymphocyte depletion—particularly of CD4 + T cells—and functional suppression, often marked by elevated inhibitory cytokines such as IL‑10, reflect a state of adaptive immune exhaustion that undermines host defense and predisposes to secondary infections and organ failure [20]. Furthermore, platelets act as crucial immune regulators; their depletion exacerbates monocyte dysfunction, leading to heightened inflammatory cytokine release (e.g., IL-6, IL-8) and contributing to immune dysregulation and worsened clinical outcomes [21].

Given these pivotal yet interconnected roles, the readily obtainable counts of neutrophils, lymphocytes, and platelets—and their derived ratios—present a compelling, yet underexplored, target for early prognostic assessment. The NLR and NPR offer the advantages of being inexpensive, rapidly available, and potentially reflective of both inflammatory and immune-coagulatory axes [11,12,15,22]. Therefore, this study sought to evaluate whether NLR and NPR, calculated from routine complete blood counts within the first 24 hours of emergency department presentation, could serve as rapid and integrative biomarkers for early risk stratification in sepsis, both individually and in combination with the established marker PCT.

Previous studies have reported that NLR may have prognostic value in septic patients [11,12], and NPR has been shown to predict mortality in infective endocarditis [15]. However, a more recent study by Schupp et al. reported that NLR was not associated with 30-day mortality in septic patients [22]. In our cohort, when analyzed as continuous variables, neither NLR nor NPR showed a significant linear association with 28-day mortality after adjusting for age, SOFA score, and Lac (Table 5). Furthermore, RCS curves (Figs 2 and 3) demonstrated no significant non-linear associations or threshold effects, as the 95% confidence intervals crossed the reference line (HR = 1) across the entire range of values for both biomarkers. These findings indicate the absence of a linear or non-linear dose–response relationship in this specific cohort.

To meaningfully compare these ratio-based indices against established inflammatory biomarkers, we first needed to identify a representative traditional marker. We evaluated three candidate markers—PCT, CRP, and WBC—by assessing their discriminative ability (C-index) alongside core clinical baseline variables (Table 4, Fig 1). The model incorporating PCT demonstrated the highest discriminative performance (C-index = 0.694, 95% CI: 0.65–0.73), supporting its selection as the reference biomarker from our cohort data. This empirical finding, coupled with the well-established role of PCT in international sepsis guidelines and its widespread validation in prognostic studies [2,23,24], provided a dual rationale for its use as the reference biomarker for subsequent comparisons with NLR and NPR.

Consistent with previous studies [25,26], the discriminative capacity of individual inflammatory markers—including PCT and CRP—remains limited. Even PCT, the most widely studied biomarker for sepsis, has a sensitivity of only 77% and specificity of 79%, and cannot be used as a single definitive test [6]. This inherent limitation underscores the need for more integrative prognostic approaches. Given the limited prognostic utility of individual markers, we hypothesized that a combination of biomarkers reflecting distinct yet complementary aspects of the host response—specifically, systemic inflammation (PCT) and cellular immune dysregulation (NLR/NPR)—could yield superior predictive performance. This approach aligns with the growing consensus that sepsis outcomes are determined by the interplay of multiple pathophysiological pathways rather than a single dominant process [27].

To test this hypothesis, we constructed two multivariable Cox models incorporating PCT and NLR (or NPR) as continuous variables with interaction terms, adjusted for LASSO-selected covariates (hypertension, SOFA score, Lac, and CRP) after confirming no severe multicollinearity (Table 7). However, as shown in Table 8, neither NLR nor NPR showed significant independent effects when added to PCT (PCT × NLR: *p* = 0.854; PCT × NPR: *p* = 0.121), nor did they provide additional prognostic value beyond PCT alone.

Several factors may explain this negative finding. According to the established understanding of sepsis immunopathology, the host immune response follows a dynamic temporal course: the early phase is dominated by hyperinflammation, whereas the immunosuppressive phase develops later in the disease process [28,29]. Our cohort was predominantly elderly (median age 81 years), and elderly patients often present with atypical symptoms [9]. Therefore, we hypothesized that combining PCT, a traditional inflammatory marker, with NLR or NPR, composite markers that may capture immune status at ED presentation, could serve as an early predictor of 28-day mortality in sepsis. However, the results were not as expected.

First, our single time-point measurement at ED presentation, regardless of the underlying disease stage, may not have consistently captured the immune information that NLR and NPR might better reflect. Second, a more scientifically sound approach would involve serial measurements over time rather than reliance on a single time-point assessment. This notion is supported by recent work demonstrating that dynamic trajectories of clinical parameters (e.g., vital signs) can identify distinct sepsis subphenotypes with different outcomes [30]. By analogy, serial measurements of NLR and NPR over time might better capture the evolving immune response and provide more accurate prognostic information than a single initial value. Third, sepsis is a highly complex pathophysiological process involving the interplay of inflammation, immune dysregulation, coagulation, and multiple organ dysfunction. Using only an infection-related marker (PCT) combined with partial immune indicators (NLR/NPR) cannot fully simulate the entire pathogenic process of sepsis. Collectively, these factors may explain the negative findings in this study.

In conclusion, neither NLR nor NPR alone, nor their combination with PCT, improved 28-day mortality prediction in this elderly cohort, this suggests that a single early measurement may be insufficient to capture the dynamic and complex pathophysiology of sepsis. Future studies should explore serial measurements over time and integrate multiple dimensions—including inflammation, immune dysregulation, coagulation, and organ dysfunction—to develop more accurate risk stratification tools. Nevertheless, given their low cost and wide availability, NLR and NPR may still hold promise as components of a multi-parameter prognostic panel, particularly in resource-limited settings.

## Limitations

This single-center retrospective study has several limitations, including the need for external validation in multi-center prospective cohorts, limited sample size for detailed subgroup analyses, inherent risks of selection bias and unmeasured confounding in retrospective data collection, exclusion of patients with hospital stay < 24 hours (estimated n < 10) which may slightly underestimate mortality but does not compromise the primary objective, exclusion of pregnant and lactating patients due to physiological leukocytosis limiting generalizability, and a predominantly elderly cohort (median age 81 years) which may limit generalizability to younger sepsis populations. Additionally, NLR and NPR are influenced by various non-infectious factors, warranting further investigation. Future studies incorporating serial biomarker measurements and clinical-microbiological stratification are needed to confirm our findings and refine risk assessment.

## Supporting information

S1 Dataset Underlying data for the study. This file contains the complete raw dataset used for all analyses, including baseline demographics, clinical characteristics, SOFA scores, Lac levels, and key parameters such as NLR, NPR, and PCT. These data support the validation of our findings and the generation of all figures and tables in the manuscript.
(XLSX)
